# Chemical Composition and *in Vitro* Antimicrobial and Mutagenic Activities of Seven Lamiaceae Essential Oils

**DOI:** 10.3390/molecules14104213

**Published:** 2009-10-20

**Authors:** Laura De Martino, Vincenzo De Feo, Filomena Nazzaro

**Affiliations:** 1 Dipartimento di Scienze Farmaceutiche, Università degli Studi di Salerno, via Ponte don Melillo, 84084 Fisciano (Salerno), Italy; E-Mail: ldemartino@unisa.it (L.D.M.); 2 Istituto di Scienze dell’Alimentazione, CNR, via Roma 64, 83100 Avellino, Italy; E-Mail: mena@isa.cnr.it (F.N.)

**Keywords:** Lamiaceae, essential oils, antimicrobial activity, mutagenic activity

## Abstract

Deeper knowledge of the potentiality of aromatic plants can provide results of economic importance for food and pharmacological industry. The essential oils of seven Lamiaceae species were analyzed by gas chromatography-mass spectrometry and assayed for their antibacterial, antifungal and mutagenic activities. Monoterpenes in the oils ranged between 82.47% (hyssop oil) and 97.48% (thyme oil), being mainly represented by oxygenated compounds. The antibacterial activity was evaluated against six pathogenic and five non-pathogenic bacterial strains. Oregano and thyme oils showed the strongest antibacterial activity against the pathogenic ones. The antifungal activity was evaluated against six fungal strains of agrifood interest: the oils tested exhibited variable degrees of activity. Two *Salmonella typhimurium* strains were used to assess the possible mutagenic activity. No oil showed mutagenic activity. Data obtained let us hypothesise that the use of essential oils could be a viable and safe way to decrease the utilisation of synthetic food preservatives. Further research is needed to obtain information regarding the practical effectiveness of essential oils to prevent the growth of food borne and spoiling microbes under specific application conditions.

## Introduction

It is well-known that most spices possess a wide range of biological and pharmacological activities [1]. These volatile compounds are widely used in cosmetics as fragrance components, in the food industry to improve the flavour and the organoleptic properties of different types of foods, and in a variety of household products. In addition to their particular flavour, many essential oils and their isolated components exhibit muscle relaxant, antibacterial and antifungal activities [2]. They are also used as carminative, expectorant, sedative, mucolytic, emmenagogue, spasmolytic, hepatoprotective, antiviral, chemopreventive remedies [3]. Particularly, the antimicrobial and antimutagenic activities of essential oils have formed the basis of many applications, including raw and processed food preservation, pharmaceuticals, and alternative medicine [4]. Restrictions imposed by competent authorities on the use of some synthetic food additives have led to renewed interest in searching for alternatives, like natural antimicrobial compounds [5]. The antimicrobial and preservative activities of spices and essential oils is well documented in many studies. For this reason, these substances have been proposed as safe and effective substitutes of synthetic preservatives [6,7,8,9]. The composition of essential oils is variable, depending on the species as well as on the parts of the plant that are utilized [10]. Most of the chemical components of essential oils are terpenoids, including monoterpenes, sesquiterpenes, and their oxygenated derivatives. These low-molecular weight and highly lipophilic compounds easily diffuse across cell membranes to induce biological reactions [11]. Previous studies demonstrated the biological activities of this class of compounds, such as the antibacterial, antifungal, antitermitic, and mosquito larvicidal properties [12]. The genotoxic activity of the essential oil of some Lamiaceae was also evaluated [1,2,13,14]. In previous papers we verified the effectiveness of some essential oils against pathogenic and non-pathogenic microbial strains [9]. In addition, we evaluated the antibacterial activity exhibited by some essential oils [15,16] as well some allelopathic properties [17]. Considering the interest of natural products for different commercial fields, a thorough knowledge of the biological and safety profile of essential oils can also give provide results of economic importance [18]. In this light, we studied the composition of the essential oils of seven species of Lamiaceae (*Ocimum basilicum* L., *Hyssopus officinalis* L., *Lavandula angustifolia* Mill., *Melissa officinalis* L., *Origanum vulgare* L., *Salvia officinalis* L., *Thymus vulgaris* L.), evaluating their antibacterial and antifungal properties against pathogenic or agrifood bacteria or fungal strains. The mutagenic property of the essential oils was also assessed.

## Results and Discussion

### Chemical composition of the essential oils

Table 1 reports the chemical composition of the investigated oils. The main constituents of *M. officinalis* essential oil were (-)-citronellal (39.56%), carvacrol (13.31%) and *iso*-menthone (8.85%); the main constituents of basil oil were *iso*-pinocamphone (35.10%) and carvone (39.70%). The compositions of balm and basil oils agree with literature data [19,20,21,22]. β-Pinene (18.20%), *iso*-pinocamphone (29.10%), and *trans*-pinocamphone (11.12%) were the most abundant components of *H. officinalis* essential oil, as reported [23]. Linalool (23.10%) and linalyl acetate (44.45%) represented the main components of the oil of *L. angustifolia*; this composition agrees with data reported in literature [24]. The main components of *O. vulgare* and *T. vulgaris* oils were *o*-cymene and carvacrol, accounting, respectively, for 41.90% and 44.01%, in oregano, and 56.20% and 24.44% in thyme oil. Moreover, the thyme oil also contains a several percentage of thymol (8.75%). The oregano oil composition appears to be in part different from others reported in literature: in fact, some papers reported *p*-cymene as the main compound of the oregano oil [25,26]. Differences were also reported for the composition of thyme oil [21,27,28]. Sage essential oil was mainly constituted by *trans*-thujone (37.95%), camphor (13.92%) and borneol (7.60%). This agrees with previous reports [29,30]. All oils tested were mainly constituted by monoterpenes, which represented a percentage ranging between 82.47%, in the hyssop oil, and 97.48%, in the oil of thyme. Among those components, oxygenated monoterpenes were in amounts ranging between 47.46% (oregano oil) and 87.59% (lavender oil). Sesquiterpenes were in lower amounts in all the oils analyzed.

### Antibacterial activity

Most of the pathogenic bacteria tested showed an appreciable sensitivity towards the essential oils used in the experiment (Table 2). *E. coli* and *B. cereus* 4384 were the most sensitive microorganisms, and, in lesser extent, *B. cereus* 4313, *E. faecalis* and *S. aureus*. On the other hand, *P. aeruginosa* was the most resistant strain, showing a low sensitivity only with the highest amounts (460 and 467 μg/paper disc) of thyme and oregano essential oils, respectively. Among the essential oils, oregano and thyme exhibited the most effective antibacterial activity against all the strains tested, in particular against *E. coli* and *B. cereus* 4384, with inhibition halos of 1.40 and 1.23 cm for *E. coli* and 1.53 and 1.43 cm for *B. cereus* 4384, respectively, when the highest doses (467 and 463 μg/paper disc of oregano and thyme essential oils, respectively) were used. Such an activity could be strictly related to their chemical composition: in fact, carvacrol, also found in balm oil, is reported for its good antibacterial effect mainly against *B. cereus* 4384, *E. coli* and *E. faecalis* [31].

Activity was also shown by lavender and sage essential oils against the same bacterial strains and with the same amount (~450 μg/paper disc), with inhibition halos ranging from 0.60 (against *B. cereus* 4313) to 0.77 cm (*E. coli*). This result could be probably related to the presence of linalool and *cis*-thujone. Basil essential oil was almost totally ineffective, exhibiting a weak antimicrobial action only against *E. coli* (0.60 cm inhibition halo), with the highest dose used (480 μg/paper disc).

The effectiveness demonstrated by all the essential oils against the pathogenic microorganism *E. coli* DSM 8579 serotype O157:H7, a pathogen of worldwide human health interest, can be considered important, as this strain produces verocytotoxins, and causes haemorrhagic colitis and haemolytic uremic syndrome. Moreover, the activity against both *B. cereus* strains could led to the use of the tested oils, except basil, as natural food preservatives against this strain, strictly linked to food-borne illnesses [32]. All Lactobacilli tested did not show any inhibition of such useful bacteria. Moreover, the available literature reports that some spices can positively influence the technological and biological performances of lactic acid bacteria, such as *Lb. sakei*, involved as starter in the manufacturing of sausages and they can improve the shelf life and safety of the final product [33]. Due to the overall activities demonstrated by oregano and thyme oils, and, in less extent, by hyssop and balm oils, their use could be useful in food technology.

**Table 1:** Chemical composition of essential oils: *Melissa officinalis* L. (Balm), *Ocimum basilicum* L. (Basil), *Hyssopus officinalis* L. (Hyssop), *Lavandula angustifolia* Mill. (Lavender), *Origanum vulgare* L. (Oregano), *Salvia officinalis* L. (Sage), *Thymus vulgaris* L. (Thyme).

Compound	Ki ^a^	Ki ^b^	Balm	Basil	Hyssop	Lavender	Oregano	Sage	Thyme	Identification ^d^
			**%^c^**	**%**	**%**	**%**	**%**	**%**	**%**	
α-Thujene	930	1035	0.12 ± 0.01	T	0.41 ± 0.02	0.23 ± 0.03	0.46 ± 0.01	0.45 ± 0.01	T	LRI, MS
α-Pinene	938	1032	0.90 ± 0.02	0.3 ± 0.01	1.05 ± 0.04	---	0.45 ± 0.03	4.44 ± 0.12	2.54 ± 0.15	LRI, MS, Co-GC
(-)-Camphene	953	1076	---	---	0.25 ± 0.04	0.67 ± 0.05	0.17 ± 0.04	4.09 ± 0.05	0.96 ± 0.07	LRI, MS, Co-GC
Sabinene	973	1132	T	0.27 ± 0.05	1.43 ± 0.90	T	T	0.44 ± 0.01	T	RI, MS, Co-GC
Hepten-3-one	975		T	---	---	---	---	---	---	LRI, MS
β-Pinene	978	1118	0.43 ± 0.06	0.46 ± 0.03	18.20 ± 0.05	---	0.21 ± 0.05	2.54 ± 0.07	---	LRI, MS, Co-GC
*cis*-Pinane	980		---	0.10 ± 0.03	---	0.11 ± 0.02	0.14 ± 0.05	---	---	LRI, MS
Verbenene	982		T	T	0.1	T	T	T	T	LRI, MS
Myrcene	993	1174	0.10 ± 0.03	0.34 ± 0.06	1.84 ± 0.20	0.34 ± 0.03	0.50 ± 0.03	0.51 ± 0.06	0.10 ± 0.02	LRI, MS, Co-GC
α-Phellandrene	995	1176	T	T	T	0.20 ± 0.03	0.15 ± 0.01	T	T	LRI, MS, Co-GC
Δ3-Carene	997	1153	---	---	---	0.27 ± 0.06	0.18 ± 0.02	---	---	LRI, MS, Co-GC
α-Terpinene	1012	1188	0.15 ± 0.08	T	0.18 ± 0.06	T	0.46 ± 0.05	T	0.11 ± 0.01	LRI, MS, Co-GC
*o*-Cymene	1020	1187	2.33 ± 0.91	0.15 ± 0.02	0.23 ± 0.02	0.65 ± 0.08	41.90 ± 0.10	2.53 ± 0.20	56.20 ± 0.20	LRI, MS, Co-GC
*p*-Cymene	1024	1280	0.60 ± 0.01	---	---	0.34 ± 0.05	0.12 ± 0.02	1.25 ± 0.11	0.15 ± 0.05	LRI, MS, Co-GC
β-Phellandrene	1029	1218	0.33 ± 0.01	0.32 ± 0.02	1.85 ± 0.20	0.12 ± 0.01	0.14 ± 0.03	0.97 ± 0.04	0.16 ± 0.08	LRI, MS, Co-GC
Limonene	1030	1203	1.44 ± 0.30	0.38 ± 0.01	1.27 ± 0.68	0.27 ± 0.02	0.35 ± 0.03	1.36 ± 0.04	0.64 ± 0.01	LRI, MS, Co-GC
1,8-Cineole	1034	1213	0.22 ± 0.00	0.55 ± 0.09	0.17 ± 0.02	T	0.61 ± 0.06	4.20 ± 0.30	T	LRI, MS
(Z)-β-Ocimene	1038	1246	T	0.10 ± 0.01	0.33 ± 0.04	1.66 ± 0.30	T	T	T	LRI, MS, Co-GC
(E)-β-Ocimene	1049	1280	T	1.21 ± 0.04	0.98 ± 0.05	0.60 ± 0.06	T	T	T	LRI, MS, Co-GC
γ-Terpinene	1057	1255	0.36 ± 0.00	T	0.18 ± 0.02	T	2.84 ± 0.20	0.10 ± 0.00	0.44 ± 0.01	LRI, MS, Co-GC
*cis*-Sabinene hydrate	1063	1556	---	---	---	0.33 ± 0.02	0.17 ± 0.03	0.15 ± 0.01	---	LRI, MS, Co-GC
*cis*-Linalool oxide	1065	1450	---	---	---	0.45 ± 0.06	---	---	---	LRI, MS, Co-GC
Fenchone	1067	1392	---	0.44 ± 0.08	---	---	---	---	---	LRI, MS
Terpinolene	1086	1265	0.13 ± 0.04	0.12 ± 0.06	0.18 ± 0.04	T	0.11 ± 0.01	T	0.66 ± 0.07	LRI, MS
Linalool	1097	1553	0.68 ± 0.06	0.70 ± 0.01	1.03 ± 0.10	23.10 ± 0.20	0.72 ± 0.30	1.10 ± 0.60	0.39 ± 0.07	LRI, MS, Co-GC
*endo*-Fenchol	1098		---	0.24 ± 0.01	---	---	---	---	---	LRI, MS
*cis*-Thujone	1105	1430	T	---	0.10 ± 0.01	T	T	---	T	LRI, MS, Co-GC
*trans*-Thujone	1115	1449	---	---	---	---	---	37.95 ± 0.80	---	LRI, MS, Co-GC
*trans*-Pinocarveol	1138	1654	---	T	0.14 ± 0.01	T	T	0.18 ± 0.03	T	LRI, MS
(-)-Citronellal	1143	1491	39.56 ± 0.40	---	---	---	---	0.22 ± 0.01	0.54 ± 0.08	LRI, MS, Co-GC
*iso*-Borneol	1144	1633	0.55 ± 0.01	---	---	---	---	---	0.12 ± 0.02	LRI, MS, Co-GC
Camphor	1145	1532	1.10 ± 0.01	0.66 ± 0.02	---	0.94 ± 0.03	T	13.92 ± 0.70	T	LRI, MS, Co-GC
Menthofuran	1150	1502	---	---	0.33 ± 0.01	---	---	---	---	LRI, MS, Co-GC
*iso*- Pinocamphone	1153	1566	T	35.10 ± 0.02	29.10 ± 0.02	0.10 ± 0.02	0.10 ± 0.02	0.10 ± 0.02	T	LRI, MS
*trans*-Pinocamphone	1159		0.44 ± 0.02	T	11.12 ± 0.90	---	T	0.27 ± 0.02	T	LRI, MS
Lavandulol	1162	1674	---	---	4.43 ± 0.40	---	---	---	---	LRI, MS
*iso*-Menthone	1163	1503	8.85 ± 0.90	---	---	---	---	---	0.11 ± 0.02	LRI, MS, Co-GC
Pinocarvone	1165	1587	T	0.43 ± 0.01	0.46 ± 0.04	T	T	T	T	LRI, MS
Borneol	1167	1719	0.15 ± 0.01	0.21 ± 0.02	0.14 ± 0.03	6.35 ± 0.94	0.26 ± 0.02	7.60 ± 0.40	0.17 ± 0.05	LRI, MS, Co-GC
Terpinen-4-ol	1176	1611	0.12 ± 0.02	0.22 ± 0.01	0.26 ± 0.06	0.25 ± 0.03	0.45 ± 0.03	0.55 ± 0.02	T	LRI, MS, Co-GC
dihydro-Carveol	1177		---	---	1.25 ± 0.15	0.36 ± 0.03	---	0.20 ± 0.01	0.22 ± 0.01	LRI, MS
*p*-Cymen-8-ol	1185	1864	T	---	T	0.28 ± 0.01	0.18 ± 0.05	0.11 ± 0.02	T	LRI, MS
α-Terpineol	1189	1706	0.11 ± 0.01	1.30 ± 0.30	1.24 ± 0.10	0.38 ± 0.05	T	0.27 ± 0.01	0.26 ± 0.04	LRI, MS, Co-GC
Myrtenal	1193	1648	---	1.01 ± 0.03	1.05 ± 0.30	0.37 ± 0.06	---	0.22 ± 0.04	0.26 ± 0.05	LRI, MS
Estragole	1195	1670	---	---	0.44 ± 0.03	---	0.11 ± 0.01	T	---	LRI, MS, Co-GC
Myrtenol	1196	1804	---	0.61 ± 0.02	1.26 ± 0.50	0.37 ± 0.03	---	0.25 ± 0.01	0.26 ± 0.04	LRI, MS
Citronellol	1213	1772	6.20 ± 0.29	---	---	---	---	---	---	LRI, MS, Co-GC
*cis*-Carveol	1226	1878	---	0.11 ± 0.01	---	---	---	---	---	LRI, MS
Carvone	1241	1752	---	39.70 ± 0.90	---	---	---	---	---	LRI, MS, Co-GC
Linalyl acetate	1248	1565	2.30 ± 0.26	0.44 ± 0.02	0.33 ± 0.01	44.45 ± 0.70	0.10 ± 0.01	1.55 ± 0.20	---	LRI, MS, Co-GC
Geraniol	1255	1857	5.70 ± 0.30	---	---	9.26 ± 0.26	---	0.33 ± 0.02	---	LRI, MS
*cis*-Anethole	1262		---	---	0.35 ± 0.05	---	---	---	---	LRI, MS
Isobornyl acetate	1277		T	T	---	0.26 ± 0.01	T	0.68 ± 0.02	T	LRI, MS
Bornyl acetate	1284	1591	T	T	T	0.24 ± 0.03	T	0.88 ± 0.04	T	LRI, MS
Cinnamic acid methyl ester	1289		---	0.11 ± 0.03	---	---	---	---	---	LRI, MS
Thymol	1290	2198	0.11 ± 0.03	---	T	---	0.75 ± 0.02	T	8.75 ± 0.90	LRI, MS, Co-GC
Carvacrol	1297	2239	13.31 ± 0.90	T	T	---	44.01 ± 0.90	0.26 ± 0.01	24.44 ± 0.90	LRI, MS, Co-GC
Myrtenyl acetate	1313		---	0.51 ± 0.03	0.56 ± 0.02	---	T	T	---	LRI, MS
Eugenol	1353	2186	0.55 ± 0.03	---	---	---	---	---	---	LRI, MS, Co-GC
Citronellyl acetate	1358	1662	1.60 ± 0.90	---	---	---	---	---	---	LRI, MS
Methyl eugenol	1369	2023	T	0.53 ± 0.03	0.67 ± 0.01	T	T	---	---	LRI, MS
α-Copaene	1377	1497	T	0.11 ± 0.01	0.15 ± 0.02	T	0.15 ± 0.04	T	T	LRI, MS
Geranyl acetate	1379	1765	1.70 ± 0.30	---	---	---	---	---	---	LRI, MS
Isoledene	1382		T	0.10 ± 0.01	0.11 ± 0.01	T	0.11 ± 0.02	T	T	LRI, MS
β-Bourbonene	1385	1535	---	1.20 ± 0.32	1.34 ± 0.30	---	---	---	---	LRI, MS
β-Elemene	1387	1600	0.60 ± 0.01	0.11 ± 0.01	T	---	T	---	T	LRI, MS
α-Gurjunene	1408	1529	0.40 ± 0.01	0.41 ± 0.04	0.55 ± 0.04	---	---	---	---	LRI, MS
Longifolene	1411	1576	0.90 ± 0.12	0.52 ± 0.01	0.53 ± 0.02	T	T	T	T	LRI, MS
β-Caryophyllene	1418	1612	0.62 ± 0.01	1.40 ± 0.50	1.00 ± 0.50	1.05 ± 0.90	0.22 ± 0.09	1.28 ± 0.03	0.15 ± 0.01	LRI, MS
β-Cedrene	1424	1638	0.34 ± 0.04	0.55 ± 0.02	0.56 ± 0.01	1.35 ± 0.15	0.56 ± 0.03	1.00 ± 0.01	---	LRI, MS
Aromadendrene	1437	1628	T	T	T	T	T	0.1	T	LRI, MS
α-Humulene	1455	1689	0.22 ± 0.01	0.55 ± 0.04	0.57 ± 0.03	0.56 ± 0.04	0.11 ± 0.02	5.90 ± 0.90	T	LRI, MS
*allo*-Aromadendrene	1463	1661	T	1.23 ± 0.50	1.36 ± 0.20	0.47 ± 0.04	T	0.11 ± 0.02	T	LRI, MS
Neryl isobutyrate	1468		---	---	---	0.10 ± 0.01	---	---	---	LRI, MS
γ-Gurjunene	1473	1687	0.22 ± 0.01	0.51 ± 0.02	0.10 ± 0.01	---	0.11 ± 0.01	0.13 ± 0.05	T	LRI, MS
*cis*-*β*-Guaiene	1490	1694	0.15 ± 0.02	---	0.44 ± 0.01	---	---	---	---	LRI, MS
Bicyclogermacrene	1491	1756	---	1.50 ± 0.02	3.10 ± 0.50	---	---	---	---	LRI, MS
*cis*-Muurola-4(14),5-diene	1510	1675	2.30 ± 0.50	3.01 ± 0.90	3.68 ± 0.90	0.29 ± 0.02	T	T	T	LRI, MS
α-7-*epi*-Selinene	1518	1740	0.64 ± 0.02	0.11 ± 0.01	0.15 ± 0.02	---	0.14 ± 0.03	0.11 ± 0.01	T	LRI, MS
Caryophyllene oxide	1580	2008	0.22 ± 0.02	---	---	0.44 ± 0.02	0.18 ± 0.02	0.77 ± 0.05	---	LRI, MS
α-Cadinol	1652	2255	0.20 ± 0.01	---	0.35 ± 0.02	---	---	---	---	LRI, MS
Total compounds			96.95	97.93	96.81	97.21	97.22	97.98	97.63	
Monoterpenes hydrocarbons			6.89	3.75	28.39	5.46	48.18	18.68	61.96	
Oxigenated monoterpenes			83.25	82.76	54.08	87.59	47.46	70.99	35.52	
Total Monoterpenes			90.14	86.51	82.47	93.05	95.64	89.67	97.48	
Sesquiterpenes hydrocarbons			6.39	11.31	13.64	3.72	1.4	7.54	0.15	
Oxigenated sesquiterpenes			0.42	0	0.35	0.44	0.18	0.77	0	
Total Sesquiterpenes			6.81	11.31	13.99	4.16	1.58	8.31	0.15	
Non terpenes			0	0.22	0.35	0	0	0	0	

The values are the mean of three replicates ± SD. ^a^: HP 5MS column; ^b^: HP Innowax column ; ^c^: LRI = linear retention index, MS = mass spectrum, Co-GC = co-injection with authentic compound; ^d^: Mass of compounds in mg/100 mg oil; t, trace (<0.05%); mean value ± standard error, *n*, three independent determinations.

**Table 2:** Inhibition of bacterial growth of essential oils: *Melissa officinalis* L. (balm), *Ocimum basilicum* L. (basil), *Hyssopus officinalis* L. (hyssop), *Lavandula angustifolia* Mill. (lavender), *Origanum vulgare* L. (oregano), *Salvia officinalis* L. (sage), *Thymus vulgaris* L. (thyme). Data are expressed in cm and do not include the diameter of paper disc. Results are shown as mean ± standard deviation (SD) of the inhibition zone (n=3).

Essential oil (µg/paper disc)	*Bacillus cereus* 4313	*Bacillus cereus* 4384	*Pseudomonas aeruginosa*	*Escherichia coli*	*Enterococcus faecalis*	*Staphylococcus aureus*
	IZ(± SD)	IZ(± SD)	IZ(± SD)	IZ(± SD)	IZ(± SD)	IZ(± SD)
**Balm 88 μg**	NA(± 0.00)	NA(± 0.00)	NA(± 0.00)	NA(± 0.00)	NA(± 0.00)	NA(± 0.00)
**Balm 177** **μg**	NA(± 0.00)	0.75(± 0.07)	NA(± 0.00)	0.65(± 0.07)	0.80(± 0.20)	NA(± 0.00)
**Balm 442** **μg**	0.63(± 0.05)	1.00(± 0.00)	NA(± 0.00)	0.67(± 0.06)	1.30(± 0.14)	0.60(± 0.05)
**Basil** **96 μg**	NA(± 0.00)	NA(± 0.00)	NA(± 0.00)	NA(± 0.00)	NA(± 0.00)	NA(± 0.00)
**Basil 192** **μg**	NA(± 0.00)	NA(± 0.00)	NA(± 0.00)	NA(± 0.00)	NA(± 0.00)	NA(± 0.00)
**Basil 480** **μg**	NA(± 0.00)	NA(± 0.00)	NA(± 0.00)	0.60(± 0.04)	NA(± 0.00)	NA(± 0.00)
**Hyssop 93** **μg**	NA(± 0.00)	NA(± 0.00)	NA(± 0.00)	NA(± 0.00)	NA(± 0.00)	NA(± 0.00)
**Hyssop 185** **μg**	NA(± 0.00)	NA(± 0.00)	NA(± 0.00)	NA(± 0.00)	NA(± 0.00)	NA(± 0.00)
**Hyssop 463** **μg**	0.63(± 0.06)	0.60(± 0.05)	NA(± 0.00)	0.77(± 0.12)	0.60(± 0.04)	0.60(± 0.00)
**Lavender 87** **μg**	NA(± 0.00)	NA(± 0.00)	NA(± 0.00)	NA(± 0.00)	NA(± 0.00)	NA(± 0.00)
**Lavender 177** **μg**	NA(± 0.00)	NA(± 0.00)	NA(± 0.00)	NA(± 0.00)	NA(± 0.00)	NA(± 0.00)
**Lavender 443** **μg**	0.63(± 0.06)	0.73(± 0.15)	NA(± 0.00)	0.73(± 0.06)	NA(± 0.00)	0.75(± 0.17)
**Oregano 93** **μg**	0.70(± 0.14)	0.9(± 0.14)	NA(± 0.00)	0.77(± 0.11)	NA(± 0.00)	NA(± 0.00)
**Oregano 187** **μg**	0.80(± 0.20)	1.00(± 0.0)	NA(± 0.00)	0.97(± 0.06)	0.83(± 0.06)	0.65(± 0.08)
**Oregano 467** **μg**	1.10(± 0.26)	1.53(± 0.06)	0.90(± 0.17)	1.40(± 0.20)	1.07(± 0.32)	0.70(± 0.00)
**Sage 91** **μg**	NA(± 0.00)	NA(± 0.00)	NA(± 0.00)	NA(± 0.00)	NA(± 0.00)	NA(± 0.00)
**Sage 183** **μg**	NA(± 0.00)	NA(± 0.00)	NA(± 0.00)	NA(± 0.00)	NA(± 0.00)	NA(± 0.00)
**Sage 457** **μg**	0.60(± 0.04)	0.63(± 0.06)	NA(± 0.00)	0.77(± 0.06)	NA(± 0.00)	0.70(± 0.40)
**Thyme 93** **μg**	NA(± 0.00)	0.80(± 0.20)	NA(± 0.00)	0.73(± 0.06)	0.70(± 0.1)	NA(± 0.00)
**Thyme 185** **μg**	NA(± 0.00)	0.87(± 0.15)	NA(± 0.00)	0.87(± 0.06)	0.73(± 0.06)	0.70(± 0.12)
**Thyme 463** **μg**	0.75(± 0.11)	1.43(± 0.06)	0.70(± 0.00)	1.23(± 0.06)	1.07(± 0.40)	0.80(± 0.0)
**Gentamycin 8 μg**	1.77(± 0.12)	1.57(± 0.12)	1.53(± 0,06)	1.57(± 0.12)	1.97(± 0.06)	0.57(± 0.12)
**Chloramphenicol 66 μg**	1.13(± 0.06)	1.37(± 0.06)	0.67(± 0.06)	1.03(± 0.06)	2.13(± 0.12)	0.83(± 0.29)
**Tetracicline 7 μg**	1.03(± 0.06)	0.83(± 0.06)	0.97(± 0.06)	1.27(± 0.12)	1.37(± 0.12)	0.57(± 0.06)
**DMSO**	NA(± 0.00)	NA(± 0.00)	NA(± 0.00)	NA(± 0.00)	NA(± 0.00)	NA(± 0.00)

NA = not active. *Lactobacillus acidophilus*, *Lactobacillus casei*, *Lactobacillus bulgaricus*, *Lactobacillus sakei*, *Lactobacillus rhamnosus*.

### Antifungal activity

We tested the action of essential oils against different species of the genus *Penicillium*, an important contaminant of foods and agricultural commodities, against one strain of *A. pullulans*, usually recognised as contaminant of vegetables [34] and against *D. hansenii*, an ubiquitous unicellular haploid yeast, commonly found in freshwater and seawater or as opportunistic parasite, in humans, fish and vegetables [35].

The results in Table 3, show that the essential oils exhibited variable degrees of antifungal activity. Generally, all oils exhibited good antifungal capabilities, in particular against *A. pullulans* and *P. simplicissimum* (at the three doses used, ranging from 87 to 480 μg/paper disc), in a dose-dependent manner. A good activity against *P. citrinum* was also recorded. Fungal growth was reduced by all essential oils, with inhibition halos ranging from 0.20 (oregano oil), to 1.43 cm (balm). This result appears of importance, being this microorganism a well known producer of the toxic metabolite citrinin, a hepatonephrotoxic mycotoxin involved in different disease outbreaks in animals and humans [36].

No activity was recorded against *D. hansenii*, except when using the highest dose of oregano and sage essential oil (inhibition halos 0.83 and 0.23 cm, respectively) and against *P. expansum* (by using 467 μg of oregano essential oil). A weak activity was detected toward *P. aurantiogriseum*, only for balm, hyssop and lavender essential oils at different doses.

Our data agree with available literature. Antifungal activity of several essential oils has been previously reported [37]. *T. vulgaris* oil was found to exhibit an inhibitory activity against *Aspergillus flavus* [38]. The antifungal activity of *H. officinalis* oil was previously tested against phytopathogenic fungi, *Pyrenophora avenae* and *Pyricularia oryzae* [39]. *S. officinalis* oil, like other essential oils, presented a broad antifungal spectrum [40,41]. The available literature reports also the antifungal activity showed by essential oils and some of their main constituents [42,43].

### Mutagenic activity

None of the essential oils tested, at any dose, showed mutagenic activity on TA98 and TA100 *Salmonella typhimurium* strains, with or without metabolic activation (Table 4). However, in previous studies, carvacrol, one of the components found in the oils with the highest antibacterial and antifungal activity, was demonstrated to be mutagenic on these strains [1]. In our experiments the non mutagenic activity of oregano essential oil could be probably due to the concomitant presence of different components which could be desmutagenic. 

It was suggested that the essential oils are a promising food preservatives, because they show antimicrobial activity at low amounts almost always without any mutagenicity [4].

Data obtained in this study clearly showed the inhibitory activity of the essential oils against several pathogenic bacterial and fungal strains. On the other hand, these oils showed nor inhibitory activity against lactic acid bacteria neither mutagenic potential. Considered together, these findings can represent an important result, in the future view to use the essential oils as natural preservatives for food products, due to their positive effect relating to safety and shelf life.

**Table 3:** Inhibition of fungal growth of essential oils: *Melissa officinalis* L. (balm), *Ocimum basilicum* L. (basil), *Hyssopus officinalis* L. (hyssop), *Lavandula angustifolia* Mill. (lavender), *Origanum vulgare* L. (oregano), *Salvia officinalis* L. (sage), *Thymus vulgaris* L. (thyme). Data are expressed in cm and do not include the diameter of paper disc. Results are shown as mean ± standard deviation (SD) of the inhibition zone (n = 3).

Essential oil (µg/paper disc)	*Penicillium simplicissimum*	*Aureobasidium pullulans*	*Penicillium citrinum*	*Penicillium expansum*	*Debaryomyces hansenii*	*Penicillium aurantiogriseum*
	IZ(± SD)	IZ(± SD)	IZ(± SD)	IZ(± SD)	IZ(± SD)	IZ(± SD)
**Balm 88 μg**	0.60(± 0.00)	0.27(± 0.06)	0.57(± 0.06)	NA(± 0.00)	NA(± 0.00)	0.30(± 0.00)
**Balm 177** **μg**	0.77(± 0.06)	0.30(± 0.00)	0.73(± 0.06)	NA(± 0.00)	NA(± 0.00)	0.33(± 0.06)
**Balm 442** **μg**	1.43(± 0.06)	0.53(± 0.12)	1.43(± 0.31)	NA(± 0.00)	NA(± 0.00)	1.00(± 0.10)
**Basil** **96 μg**	0.60(± 0.00)	0.13(± 0.23)	0.07(± 0.12)	NA(± 0.00)	NA(± 0.00)	NA(± 0.00)
**Basil 192** **μg**	0.77(± 0.06)	0.37(± 0.06)	0.20(± 0.17)	NA(± 0.00)	NA(± 0.00)	NA(± 0.00)
**Basil 480** **μg**	1.13(± 0.12)	0.67(± 0.06)	0.90(± 0.10)	NA(± 0.00)	NA(± 0.00)	NA(± 0.00)
**Hyssop 93** **μg**	0.60(± 0.00)	0.43(± 0.06)	0.50(± 0.00)	NA(± 0.00)	NA(± 0.00)	0.27(± 0.06)
**Hyssop 185** **μg**	0.77(± 0.6)	0.53(± 0.06)	0.60(± 0.00)	NA(± 0.00)	NA(± 0.00)	0.30(± 0.00)
**Hyssop 463** **μg**	1.03(± 0.06)	0.80(± 0.00)	0.73(± 0.06)	NA(± 0.00)	NA(± 0.00)	0.27(± 0.06)
**Lavender 87** **μg**	0.57(± 0.06)	0.40(± 0.00)	0.43(± 0.06)	NA(± 0.00)	NA(± 0.00)	NA(± 0.00)
**Lavender 177** **μg**	0.77(± 0.06)	0.53(± 0.06)	0.60(± 0.00)	NA(± 0.00)	NA(± 0.00)	NA(± 0.00)
**Lavender 443** **μg**	1.17(± 0.06)	0.90(± 0.10)	0.87(± 0.12)1 0.12)	NA(± 0.00)	NA(± 0.00)	0.07 (± 0.06)
**Oregano 93** **μg**	1.00(± 0.00)	0.57(± 0.06)	0.20(± 0.17)	NA(± 0.00)	NA(± 0.00)	NA(± 0.00)
**Oregano 187** **μg**	1.20(± 0.00)	0.70(± 0.00)	0.47(± 0.12)	NA(± 0.00)	NA(± 0.00)	NA(± 0.00)
**Oregano 467** **μg**	1.50(± 0.00)	1.10(± 0.17)	0.57(± 0.12)	0.07(± 0.06)	0.83(± 0.06)	NA(± 0.00)
**Sage 91** **μg**	0.50(± 0.00)	NA(± 0.00)	0.50(± 0.00)	NA(± 0.00)	NA(± 0.00)	NA(± 0.00)
**Sage 183** **μg**	0.73(± 0.06)	0.27(± 0.15)	0.37(± 0.32)	NA(± 0.00)	NA(± 0.00)	NA(± 0.00)
**Sage 457** **μg**	1.07(± 0.12)	0.23(± 0.06)	0.90(± 0.10)	NA(± 0.00)	0.23(± 0.06)	NA(± 0.00)
**Thyme 93** **μg**	0.77(± 0.06)	0.93(± 0.06)	0.57(± 0.06)	NA(± 0.00)	NA(± 0.00)	NA(± 0.00)
**Thyme 185** **μg**	0.87(± 0.06)	1.07(± 0.12)	0.80(± 0.10)	NA(± 0.00)	NA(± 0.00)	NA(± 0.00)
**Thyme 463** **μg**	1.60(± 0.40)	1.47(± 0.06)	1.17(± 0.15)	NA(± 0.00)	NA(± 0.00)	NA(± 0.00)
**DMSO**	NA(± 0.00)	NA(± 0.00)	NA( ± 0.00)	NA( ± 0.00)	NA( ± 0.00)	NA( ± 0.00)

NA= not active.

**Table 4:** Mutagenicity of essential oils: *Melissa officinalis* L. (balm), *Ocimum basilicum* L. (basil), *Hyssopus officinalis* L. (hyssop), *Lavandula angustifolia* Mill. (lavender), *Origanum vulgare* L. (oregano), *Salvia officinalis* L. (sage), *Thymus vulgaris* L. (thyme). Data are expressed as number of his+ revertant colonies/plate (mean ± standard deviation -SD-) in strains *S. typhimurium* TA98 and TA100 in presence (+S9) and absence (-S9) of the metabolic activation.

Essential oil	TA98 - S9	TA100 - S9	TA98+S9	TA100 +S9
	CFU/Plate ( ± SD)	CFU/Plate( ± SD)	CFU/Plate ( ± SD)	CFU/Plate ( ± SD)
**Balm 88 μg**	46.00(± 8.00)***	229.00(± 12.17) ***	53.00(± 6.56) **	393.00(± 14.73) *
**Balm 177** **μg**	47.67(± 5.86) ***	220.00(± 7.37) ***	50.33(± 5.03) **	352.67(± 11.24) **
**Balm 442** **μg**	40.33(± 8.39) ***	175.00(± 29.82) ***	58.67(± 7.77) **	378.33(± 17.56) **
**Basil** **96 μg**	52.67(± 4.62) **	209.00(± 61.88) ***	82.00(± 13.11) ***	81.33(± 18.04) **
**Basil 192** **μg**	42.67(± 13.61) ***	217.00(± 27.87) ***	62.67(± 20.03) ***	83.00(± 8.19) **
**Basil 480** **μg**	53.67(± 6.03) ***	156.67(± 25.32) ***	55.33(± 8.08) ***	110.67(± 21.01) **
**Hyssop 93** **μg**	26.67(± 6.66) ***	256.33(± 22.59) ***	40.67(± 2.52) ***	357.00(± 24.02) **
**Hyssop 185** **μg**	32.67(± 7.51) ***	226.00(± 26.91) ***	61.33(± 12.86) ***	293.33(± 91.09) *
**Hyssop 463** **μg**	35.00(± 5.00) ***	165.00(± 54.06) ***	56.33(± 4.73) ***	168.50(± 41.72) **
**Lavender 87** **μg**	24.00(± 1.00) ***	107.33(± 6.81) ***	55.67(± 9.07) ***	90.00(± 27.84) **
**Lavender 177** **μg**	41.33(± 7.77) ***	115.33(± 16.17) ***	39.67(± 8.96) ***	170.00(± 60.92) **
**Lavender 443** **μg**	19.67(± 8.08) ***	103.67(± 13.87) ***	73.33(± 15.28) ***	255.00(± 5.00) *
**Oregano 93** **μg**	30.33(± 4.73) ***	170.00(± 43.59) ***	74.00(± 4.00) ***	277.00(± 12.12) ***
**Oregano 187** **μg**	41.33(± 7.23) ***	174.00(± 48.99) ***	77.33(± 11.37) ***	342.00(± 92.37) *
**Oregano 467** **μg**	20.00(± 2.00) ***	121.00(± 8.08) ***	56.00(± 8.72) ***	308.33(± 64.22) *
**Sage 91** **μg**	54.00(± 6.00 ) ***	246.00(± 5.29) ***	63.33(± 14.43) ***	345.33(± 8.08) **
**Sage 183** **μg**	45.33(± 2.52) ***	250.00(± 9.17) ***	45.00(± 7.55) ***	310.00(± 14.14) *
**Sage 457** **μg**	50.33(± 4.73) ***	203.00(± 20.82) ***	64.67(± 5.03) ***	310.33(± 26.84) **
**Thyme 93** **μg**	48.33(± 7.77) ***	212.00(± 13.86) ***	55.00(± 4.36) ***	292.33(± 10.69) ***
**Thyme 185** **μg**	30.33(± 6.81) ***	193.00(± 12.22) ***	60.33(± 8.14)	368.67(± 10.26) **
**Thyme 463** **μg**	25.67(± 1.15) ***	214.00(± 22.50) ***	47.00(± 3.61)	353.33(± 5.77) **
**SR**	37.00(± 4.10)	170.46(± 55.77)	48.50(± 4.51)	245.00(± 47.00)
**PC**	555.33(± 389.39)	1108.58(± 356.67)	159.87(± 55.31)	562.50(± 74.40)

CFU: colony forming units; SR: spontaneous revertants used as negative control; PC: positive control, in presence of standard mutagen agents: for TA 98 (-S9), *o*-nitrofluorene (1 µg/plate); for TA100 (-S9), sodium azide (5 µg/plate); for TA98 (S9) and TA100 (S9), 2-aminoantracene (5 µg/plate); * p < 0.05; ** p < 0.01; *** p < 0.001 vs positive control.

## Experimental

### Essential oils

Essential oils from the aerial parts of *Ocimum basilicum* L. (basil), *Hyssopus officinalis* L. (hyssop), *Lavandula angustifolia* Mill. (lavender), *Melissa officinalis* L. (balm), *Origanum vulgare* L. (oregano), *Salvia officinalis* L. (sage), *Thymus vulgaris* L. (thyme), were purchased from Azienda Chimica E Farmaceutica (A.C.E.F.) Spa (Fiorenzuola d´Arda, Italy). The density of the oils was furnished by A.C.E.F., as follows: *Ocimum basilicum* L. (0.954 g/mL), *Hyssopus officinalis* L. (0.926 g/mL), *Lavandula angustifolia* Mill. (0.893 g/mL lavender), *Melissa officinalis* L. (0.883 g/mL), *Origanum vulgare* L. (0.937 g/mL), *Salvia officinalis* L. (0.919 g/mL), *Thymus vulgaris* L. (0.927 g/mL).

### Chemical characterisation

The oils were analysed by Gas Chromatography (GC) and Gas Chromatography-Mass Spectrometry (GC-MS). GC analyses were carried out using a Perkin-Elmer Sigma-115 gas chromatograph with a data handling system and a FID. Separation was achieved by using a fused-silica capillary column HP-5 MS, 30 m length, 0.25 mm internal diameter, 0.25 μm film thickness. The operating conditions were as follows: injector and detector temperatures, 250 °C and 280 °C, respectively; oven temperature programme: 5 min isothermal at 40 °C, subsequently at 2 °C/min up to 250 °C and finally raised to 270 °C at 10 °C min^-1^. Analysis was also run by using a fused silica HP Innowax polyethylene glycol capillary column (50 m × 0.20 mm i.d., 0.20 µm film thickness). In both cases helium was used as the carrier gas (1 mL/min). Diluted samples (1/100 v/v, in *n* hexane) of 1 μL were injected manually at 250 °C, and in the splitless mode.

GC-MS analysis was performed using an Agilent 6850 Ser. A apparatus, equipped with a fused silica HP-1 capillary column (30 m × 0.25 mm i.d.; film thickness 0.33 μm), linked on line with an Agilent Mass Selective Detector MSD 5973; ionization voltage 70 electron multiplier energy 2,000 V. Gas chromatographic conditions were as given above, transfer line was kept at 295 °C. Most components were identified from their GC retention indices, with either those reported in literature [44,45] or with those of authentic compounds available in our laboratories, purchased from Sigma Aldrich, Co, Milan, Italy. The retention indices were determined in relation to a homologous series of *n*-alkanes (C_8_-C_24_) under the same operating conditions. Further identification was made by comparison of their MS spectra on both columns with either mass spectra stored in NIST 02 and Wiley 275 libraries or with mass spectra from the literature [44,46] and our homemade library. Component relative concentrations were calculated based on GC peaks without using correction factors. The percentage composition of the oils was computed by the normalization method from the GC peak areas, calculated as mean values of three injections from each oil, without using correction factors.

### Antibacterial activity

The inhibition halos test on agar plate was employed to investigate the antibacterial activity of essential oils. The samples were tested against the following bacteria: non-pathogenic strains (*Lactobacillus acidophilus* DSM 20079; *Lactobacillus casei* DSM 9595; *Lactobacillus bulgaricus* DSM 20081; *Lactobacillus sakei* DSM 20494; *Lactobacillus rhamnosus* DSM 20711); pathogenic Gram positive strains *Bacillus cereus* (DSM 4313 and DSM 4384), *Staphylococcus aureus* DSM 25923 and *Enterococcus faecalis* DSM 2352; Gram negative strains, *Escherichia coli* DSM 8579 and *Pseudomonas aeruginosa* (ATCC 50071). All strains were purchased by Deutsche Sammlung von Mikroorganismen und Zellkulturen GmbH (DSMZ Germany). Each strain was incubated at 37 °C for 18 h into own specific growth medium: Lactic Acid Bacteria were grown in Man de Rogosa Sharpe (MRS) broth (Oxoid), *E. coli*, *E. faecalis*, *S. aureus*, *P. aeruginosa* and *B. cereus* in Nutrient Broth (Oxoid). The microbial suspensions (1× 10^8^ Colony Forming Units-CFU-/mL) were uniformly spread onto the specific solid media plates (Ø = 90 mm dishes). Sterile Whatman n° 1 paper filter discs (Ø = 5 mm) were individually placed on the inoculated plates and impregnated with different amounts of essential oils, previously diluted 1:10 (v/v) in dimethylsulfoxide (DMSO) (final amount ranging from 88 to 490 μg/paper disc). The exact amounts of each oils are reported in Table 2. After 30 min under sterile conditions at room temperature, plates were incubated at 37 °C for 24–48 h, depending on the strain. The diameter of the clear zone shown on plates was accurately measured and the antibacterial activity expressed in cm (not including disc diameter of 0.5 cm). Sterile deionised water and DMSO (10 µL/paper disc) were used as negative control. Gentamycin (8 µg/paper disc), chloramphenicol (66 µg/paper disc) and tetracycline (7 µg/paper disc), in physiological solution, served as positive controls. Samples were tested in triplicate and results expressed as mean ± standard deviation. 

### Antifungal activity

The inhibition halos test on agar plate was employed to investigate the antifungal activity of the essential oils. Six fungal strains of agro-food interest (*Penicillium citrinum* DSM 1997, *Penicillium simplicissimum* DSM 1097, *Aureobasidium pullulans* DSM 62074, *Penicillium expansum* DSM 1994, *Penicillium aurantiogriseum* DSM 2429, *Debaryomyces hansenii* DSM 70238), were used. All strains were purchased by DSMZ. Sterile Whatman n° 1 paper filter discs (Ø = 5 mm) were individually placed on the inoculated plates (Ø = 90 mm dishes) and impregnated with different amounts of essential oils previously diluted 1:10 (v/v) in DMSO (final amount ranging from 88 to 490 μg/paper disc), were used. The exact amounts of each oils are reported in Table 3. A cell suspension of fungi was prepared in sterile distilled water adjusted to contain approximately 10^6^ CFU/mL, and plated onto Potato Dextrose Agar (PDA) (Oxoid). After 20 min under sterile conditions at room temperature, plates were incubated at 28 °C for 48-72 h. When the mycelium of fungi reached the edges of the control plate (negative control without the samples added extracts), the diameter of the clear zone shown on plates was accurately measured and the antifungal activity expressed in cm (not including disc diameter of 0.5 cm). DMSO was used as negative control (10 µL/paper disc). Samples were tested in triplicate and the results are expressed as mean ± standard deviation.

### Mutagenic activity

For mutagenicity assay, the bacteria reverse mutation assay (Ames test) plate incorporation method, was performed [47] by using the *Salmonella typhimurium* histidine-requiring strains TA98 and TA100, purchased from Molecular Toxicology Inc. (Moltox™, Annapolis, MD, USA). Strains were aerobically allowed to grow overnight at 37 °C in Nutrient Broth n°2 (Oxoid) supplemented with ampicillin. With the aim to define the amount of the essential oils that did not give cytotoxic effect, a preliminary test was performed by up to 900 μg/plate of each oil. The toxicity was evaluated by the complete absence of background lawn [48]. The test was carried out by addition to 1.8 mL of molten agar of the following: 0.1 mL of the overnight bacterial culture, different amounts of essential oils previously diluted 1:10 (v/v) in DMSO (final amount ranging from 88 to 490 μg), 0.5 mL phosphate buffer (0.1 M, pH 7.4) or 0.5 mL of the metabolic activation S9 mixture. The exact amounts of each oil are reported in Table 4. The mixture was immediately plated onto minimal medium agar plates (Ø = 90 mm dishes) (previously added with 0.2 mL of sterile 0.5 mM Bio-His) and incubated at 37 °C for 48 h in the dark. The rat liver post-mitocondrial supernatant fraction (S9) [47,48] induced by a polychlorinated biphenyl mixture, Aroclor 1254 (0.7 nmol cytochrome P450/mg protein), in male Sprague-Dawley rats, used for the indirect test, was purchased from Moltox™ (Annapolis, MD, USA). As positive controls, the direct acting mutagens *o*-nitrofluorene (1 μg/plate) was used for strain TA98 and sodium azide (5 μg/plate) was used for strain TA100; the indirect acting mutagen 2-amino-antracene (5 μg/plate) was used for both TA98 and TA100 stains. DMSO (10 µL/plate) was used as negative control. Samples were tested in triplicate and the results are expressed as mean ± standard deviation. 

### Statistical analysis

Data were ordered in homogeneous sets, and the Student’s *t* test of independence was applied [49].

## Conclusion

Data obtained showed the inhibitory activity of the essential oils assayed against pathogenic bacterial and fungal strains. On the other hand, these oils showed no inhibitory activity against lactic acid bacteria. These findings, considered together, can represent an important result, in the future view to use the essential oils as natural preservatives for food products, due to their positive effect on their safety and shelf life. 
